# Integrated early childhood development policy in Iran: a qualitative policy process analysis

**DOI:** 10.1186/s12889-021-10646-7

**Published:** 2021-04-02

**Authors:** Omolbanin Atashbahar, Ali Akbari Sari, Amirhossein Takian, Alireza Olyaeemanesh, Efat Mohamadi, Sayyed Hamed Barakati

**Affiliations:** 1 Department of Health Management and Economics, School of Public Health, Tehran University of Medical Sciences, Tehran, Iran; 2 National Institute of Health Research, TehranUniversity of Medical Sciences, No. 70, Bozorgmehr Ava., Vesal St., Keshavars Blvd., Tehran, 1416833481 Iran; 3 Health Equity ResearchCenter (HERC), TehranUniversity of Medical Sciences, Tehran, Iran; 4 Department of Global Health and Public Policy, School of Public Health, Tehran University of Medical Sciences, Tehran, Iran; 5 Population, Family and School Health Office, Ministry of Health and Medical Education, Tehran, Iran

**Keywords:** Early childhood, Integrated development, Policy analysis, Policymaking process

## Abstract

**Background:**

Integrated early childhood development (IECD) is a comprehensive approach to optimal development of children in different developmental domains from fetal stage to eight years of age. The aim of this study was to identify the factors affecting the process of policy-making for early childhood development and to clarify how these factors affect decision-making and create challenges in this regard.

**Method:**

In a qualitative study, we used two main data sources including document analyses and interviews. Using purposive sampling, forty semi-structured interviews with policymakers and informants in the fields related to children were conducted in Tehran from October 2017 to June 2018. Also, 62 national and 10 international relevant documents were reviewed. A deductive-inductive approach was used to analyze the data. We used the MAXQDA11 software for data management.

**Results:**

we identified 13 themes and 29 subthemes related to the stages of policymaking process including: Agenda setting (problem stream, policy stream, politics stream), Policy formulation (formulation and approval process, policy sustainability, mechanisms of stakeholders’ participation in policymaking), Policy implementation (conceptual ambiguity, intersectoral and trans-sectoral issues, structural capacities, mobilization of resources), and Policy evaluation (continuous and routine data registry system, comprehensiveness of indexes). We propose 19 policy recommendations to improve the situation.

**Conclusion:**

As a multidisciplinary and multi-sectoral field with different domains, early childhood development (ECD) requires a more active role on the part of policymakers in governmental levels in supporting the related policies. Unless policymakers change their approach to decrease nonintegrated and non-comprehensive policymaking for ECD, child development will be compromised, which endangers the eventual sustainability of the society since improved IECD policy-making process improves developmental outcomes in children. In this regard, attention should be paid to the role of reinforcing intersectoral collaboration through incorporating it in the missions and the evaluation items of organizations, creating commitment in high organizational levels, and developing an inter-ministerial policymaking framework that clearly specifies the roles and responsibilities of every single sector and their interactions and collaborations.

**Supplementary Information:**

The online version contains supplementary material available at 10.1186/s12889-021-10646-7.

## Background

Integrated early childhood development (IECD) is a comprehensive approach to children from conception to eight years of age as well as to their parents and caregivers who have a significant contribution to the children’s social, emotional, cognitive, verbal, mental, and physical growth and development. IECD policymaking includes all services and strategies that provide primary health care, proper nutrition, education, nurture and motivation in care, and a safe and clean environment for children and their families. Therefore, IECD requires multi-sectoral collaboration if children’s needs are to achieved [1]. Cohesive ECD originated from the simple and convincing idea of comprehensive care, which avoided segmentation, gathered all sectors and stakeholders around a common mission, and provided the best start for all girls and boys. However, delivering this simple and convincing idea may be very complicated and contentious depending on the financial and political background and capacity for reform, available infrastructures, priorities of donors and NGOs, and some other variables [2].

Given the importance of ECD, including the high rate of return in investment in early childhood [3] and rapid brain development in this period [4], a number of countries have designed and implemented cohesive ECD policies to utilize the benefits of early childhood as the most cost-efficient period of life thereby investing in human capital development and sustained socioeconomic development [5]. Better decisions and policies help to achieve better outcomes in children’s health indices, in reducing inequality, and in improving children’s developmental outcomes in different developmental domains [6].

Although many efforts have recently been made to improve children’s health in Iran, informants and executive authorities believe that the current status of Iran, in terms of normal development of children and the related activities, faces many challenges, [7] and that some development-related problems prevent children from enjoying their rights. One of the most important challenges is the lack of a holistic and comprehensive view of child development and the low levels of social awareness [8]. In addition, another major problem impeding early childhood development programs in Iran is the lack of a national plan for this age range as well as implementation defects to the effect that none of the previous development plans have paid attention to this area. Therefore, governmental organizations have not had, and still do not have, any programs or instructions in this regard [7].

Hence, with regards to the importance of children’s age group and the country’s conditions, the Ministry of Health offered an initial proposal for formulating a national document for IECD in 2008 [9]. However, this document has not been implemented at a national level after several years and is still viewed as a pilot project. To continue this activity, it is of paramount importance to sensitize legislating and policy-making bodies to formally support and implement IECD programs.

This study was done for the first time in Iran. In other words, no other studies have so far been conducted in this country to investigate ECD policies. In other countries, however, there are many studies that have analyzed policies in one or more areas of ECD. For example, the studies by Kang’ethe (2017) [10] and Tsegay (2017) [11] analysed education policies in Kenya and China, respectively. Binagwaho’s study in Rwanda [12] analyzed health and social policies in this country, and that of Nichols (2008) in Australia [13] analyzed human services including health and education policies from the perspective of ECD. Based on the gap in the existing literature, this study was conducted to explain and analyze the ECD policy-making process in Iran. The study also seeks to answer the question of how the ECD policy should be put on the agenda, formulated, adopted, implemented and evaluated. Moreover, the study aims to identify the factors affecting the ECD policy process and to clarify the effects of these factors on decision making and the challenges in this process. The findings of the present study can contribute to human capital development from early years and can strengthen the contribution of ECD to sustainable development.

## Methods

### Study design and data collection

The data of this exploratory qualitative study were collected through a review of the documents as well as in-depth interviews. In the current study, attempts were made to identify the IECD policy-making process. The process is described as the way in which policies are initiated, developed or formulated, negotiated, communicated, implemented and evaluated [14].

The stages of the policymaking process and the theories which are applied to their analysis are presented in the following:

**Agenda setting** is a process in which the problems and alternate solutions gain or lose the attention of the public and decision-makers [15]. Kingdon’s multiple streams framework was used to analyze this stage. It describes policymaking in terms of problems, policies, and political streams [16].

**Policy formulation** is the first stage in the policymaking process, which has two basic stages: determining the policy options and selecting the preferred option [17].

**Policy implementation** begins once the policies are passed and enacted. This stage includes the questions “who, what, and when” with regard to creating an operational plan [18]. In this study, the interactive model of policy implementation, which is a combination of top-down and bottom-up approaches, was used for policy implementation analysis. This model was proposed by Grindle and Thomas in 1991, which is in contrast with the linear model of policy implementation [15].

**Policy evaluation** is an activity through which the value, efficiency, and feasibility of a policy is perceived [19].

### Data collection

#### Documents analysis

The policy document designed for ECD in Iran (abbreviated as TAK in Persian) and other relevant documents in different fields including health, nutrition, early care and education, and support were analyzed from 1964 to 2018. Because there were not many policy documents in some areas such as early care and education, and social support for children in the country or even some important policies such as the “Universal Preschool Education Policy of Iran 1964 “ or “The International Declaration on the Rights of the Child 1959” were adopted many years ago, the researchers decided to consider a longer span of time to search for relevant policy documents. In this regard, we searched laws, national upstream documents, programs and reports developed and implemented by the government, the Ministry of Health and Medical Education, the Social Welfare Organization, the Ministry of Cooperatives, Labor, and Social Welfare, the Ministry of Education, the Parliament, the Judicial System of Iran and other organizations involved in ECD. We used different search methods, including searching the websites of the mentioned organizations, the literature, and the personal and organizational relationships to identify and access these documents. For identifying international documents, we searched the websites of the international organizations such as World Health Organization and UNICEF and the literature in journals and data bases including Google scholar, Science direct, BMJ journals, Emerald, web of science, PubMed and Lancet with selected key words including early child OR young children, develop OR integrated grows, international policy OR program. Among the national and international identified documents, we included the documents that targeted a wider range of 0 to 8-year-old children and were implemented at the national level. It should be noted that due to the high volume of civil laws, these laws have not been analyzed.

In other hand, we used criteria of Scott method for evaluating the quality of the documents including authenticity, credibility, representativeness (representative of all the documents in that category) and meaning (what they say) [20]. Also, source of communication of the document was considered (which official and government sources were considered). Finally, 64 national and 10 international documents were analyzed.

#### Interviews

Face-to-face, in-depth, semi-structured interviews were conducted using an interview guide (Appendix 1). Before the interviews, necessary information regarding the study and its objectives were given to the participants and informed consent was obtained from them verbally. Moreover, they were assured that their information would remain confidential and the data of the study would be analyzed anonymously. It is worth mentioning that the current study has been confirmed by the Ethical Committee of Tehran University of Medical Science and all the used methods were performed in accordance with the relevant guidelines and regulations.

### Sampling method and setting

In the second phase, purposive sampling approach with maximum variation in terms of scientific background, activity domain, employment status, gender and executive experience was considered. In addition, the snowball sampling method was used to identify more interviewees. The participants were divided into five groups, including policymakers (PM), managers (M), academics and researchers (Aca), NGOs’ representatives (NGO-R), and children service providers (CSP) from different organizations related to ECD (Ministry of Health and Medical Education, State Welfare Organization, Ministry of Education, Ministry of Cooperatives, Labor, and Social Welfare, Ministry of Justice, Children’s Medical Center, The Islamic Consultative Assembly, Society for Protecting the Rights of the Child (SPRC), universities and research centers, etc.). The participants met at least one of the following criteria:

- Specializing in majors related to children or neuroscience, social sciences, human sciences, and rehabilitation sciences
- Having at least three years of professional experience with children in non-governmental or governmental sectors
- Having a position related to children’s affairs in non-governmental or governmental sectors at the time of the study
- Having knowledge of macro policies related to children.

Finally, forty interviews, each lasting for 30–90 min, were conducted from October 2017 to June 2018 in Tehran, Iran. (Appendix 2) Among the participants, 57.5% were male and 42.5% were female. Also, 25% were in the age group of 50–60 years, 35% 40–50 years, 15% 30–40 years, 7.5% 60–70 years, and 17.5% 70–80 years.

### Data analysis

First, the qualitative content analysis was used to develop an information worksheet to collect and categorize selected documents (Appendix 3) and to prepare them for further analysis. Then, a deductive-inductive approach was used to analyze the data obtained from the documents information worksheet and interviews. For this purpose, the interviews were transcribed verbatim. The researchers became familiarized with the data through listening to audiotaped interviews, transcribing the interviews, and reviewing the notes taken during the interviews. Then, codes were extracted from the summaries of interviews, the open coding was carried out, and the extracted codes were categorized based on the stages of the policymaking process. Coding and data categorization were done manually using the MAXQDA11 software simultaneously.

## Results

The results of this study are presented based on the stages of the policy process as analytical categories including agenda setting, policy development, policy implementation, and evaluation. In our study, 13 themes and 29 subthemes were identified. (Table 1) These categories are presented in the following:

**Table 1:** Policy “process” Analysis of IECD

1. Factors affecting agenda setting for IECD			
**Theme**	**subtheme**	**Examples**	**Relation**
1.1- **Problem stream**	Appreciating the need for a multifaceted, integrated, and justice-oriented approach to child development	-Lack of emphasis on eliminating the existing inequalities. - Lack of focus on all aspects of children’s development - Lack of integration and coordination between various policies and programs	(PM13) (CSP23) (M 27)
	Ignoring child-related affairs compared to other public issues	- Lack of serious attention to child-related issues - Low priority of ECD compared to other programs	(PM21) (CSP16)
	Children’s inability to speak their needs and rights	-Ignoring children’s rights and needs -Need for a certain body to take care of child-related issues	(M7) (Aca3)
1.2- **Politics stream**	Increased scientific evidence for the importance of IECD	-Understanding the importance of ECD by some policymakers -Considering numerous studies and reports in the world -Indicating the need for investing in ECD, including the Heckman study or papers published in the Lancet.	(PM32)
	International political movements	-Addressing equity from the start according to the recommendations for closing the gap in a generation. -International organizations’ instructions and objectives regarding the rights of the child and ECD	(PM8)
	National political movements	- Referring to ECD in the Sixth Development Plan -Establishing the Committee on Social Determinants of Health and designating ECD as a priority	(PM30) (PM8)
1.3- **Policy stream**	Designing a national policy for IECD	-Assembling committees in different fields to prepare reports on children’s status -Establishing the IECD secretariat in the Ministry of Health	(PM12) (PM13)
2. **Factors affecting formulation of IECD policies**			
**Theme**	**Subthemes**	**Examples**	**Relation**
2.1- **Formulation and approval process**	Lengthy and time-consuming administrative procedures for formulating and approving policies	-Spending a long time on planning and formulating IECD documents -Expiration of policies before implementation -Slow pace of processes; -Current policies not being up-to-date due to long intervals between formulation and implementation	(PM25) (NGO-P 28)
	Perfectionism and duplication in formulating programs	-Spending a long time on, formulating and duplicating programs and documents -Presence of several documents and programs regarding IECD with different titles	(PM32) (PM30)
2.2- **Policy sustainability**	Policies changes as a result of changing the managers	-Differences in prioritization -Differences in adopted solutions for addressing problems -Failure to achieve the intended approach in the ECD program -Lack of belief in activities with long-term results and focusing on fast-yielding activities due to short management life	(Aca 24) (M6) (PM8)
2.3- **Mechanisms regarding the participation of stakeholders in policymaking**	Lack of participation from other organizations in policy formulation	-Other organizations having no feeling of belonging to programs -Emergence of tensions between organizations and lack of opportunity for optimal collaboration in implementing policies and programs	(M 27) (PM19)
	Inappropriate composition of the Supreme Council of Health Policy	-Not using different specializations like sociology, psychology, economic, etc. for policymaking and decision-making	(Aca3)
	Using people sharing similar views with the Ministry of Health in related committees	-Removing individuals with opposite views from related committees; -Lack of attention to opposite views in related committees -Unwillingness to suggest opposite ideas for fear of being removed from the related committees	(PM15)
3. **Factors affecting implementation of ICED policies**			
**Theme**	**Subtheme**	**Examples**	**Relation**
3.1- **Conceptual ambiguity**	Different interpretations and lack of consensus on the concept of ECD	-Creating problems due to incorrect understanding of the concept of ECD -Resistance against ECD due to considering it a western concept	(PM19) (Aca26)
3.2- **Intersectoral issues**	Intersectoral competition	-Poor intersectoral collaboration -Conflicts over stewardship -Resistance against the leading or coordinating role of a same-level organization and believing in supervision and leadership from higher levels -Inconsistency between the Ministry of Health, the Ministry of Education, and the Ministry of Social Welfare. -Pursuing benefits of organization or union. -Weak inter-sectoral collaboration due to cultural context.	(CSP23) (Aca 24) (PM10) (NGO-P 11) (PM 19)
	Inefficiency of intersectoral collaboration in the form of intersectoral agreements	-Organizations’ lack of commitment to intersectoral agreements due to lack of executive requirements	(PM8)
3.3- **Trans-sectoral issues**	Lack of political commitment	-Lack of adequate support from high-level managers for implementing the Document -Lack of executive will due to lack of support from the program in high governmental levels -Lack of commitment in top level managers and convening few meetings in top echelons of organizations in this regard	(PM8) (PM22) (M14)
	Lack of a supreme body or structure for monitoring and coordinating childhood development programs	-Sameness of supervising and executive bodies in children-related issues -Lack of accountability in organizations involved in children’s issues for their programs -Lack of coordination between different organizations involved in children’s issues and problems resulting from lack of coordination including parallel works, waste of resources, contrasting programs, and lack of a comprehensive data bank for children’s data in different areas	(PM31) (PM36)
	Legal issues	-Inappropriate laws for children and the violation of their rights -Different interpretations of laws resulting in confusion in implementing them -Decreased commitment of organizations to enforcing laws -Presence of legal gaps in some areas related to children	(PM38) (NGO-P2) (CSP35) (PM31)
3.4- **Mobilization of resources**	Shortage of skilled service providers in some fields of ECD	-High workload of healthcare staff for accepting new responsibilities like screening programs -Lack of attention to standards in hiring manpower in kindergartens in terms of numbers of staff and skills -Lack of trained manpower in schools as providers of healthcare and development services -Lack of skilled manpower in some parts of the country in fields of counselling, child psychology, social work, etc. -Absence of a curriculum related to ECD in the education of child-related service providers	(PM10) (PM13) (CSP18) (M14)
	Inadequate budget and improper budget allocation	-Not allocating sufficient budget to ECD due to the low share of health sector and the low share of preventive measures in the budget. -Developmental screening in one age range due to severe shortage of financial resources for developmental screening -Inability to implement efficient educational programs about child development due to the shortage of resources -Insufficiency of allocated budget relative to the total number of children suffering from malnutrition	(M17) (CSP30) (PM10) (M17)
	Lack of insurance coverage for some services like rehabilitation for developmental disorders, special diseases, counselling, etc. for children	-Increased exposure of families to catastrophic health expenditures -Lack of involvement of specialists working in fields related to children in the diagnosis and treatment of developmental disorders due to lack of insurance coverage and similarity of fees	(PM21) (PM 15)
3.5- **Structural capacities**	Unavailability of required infrastructures	-Shortage of diagnostic and rehabilitative facilities and equipment for developmental disorders -Lack of standard educational environments in kindergartens and primary schools -Unavailability of Kindergarten services for all children -Lack of structures for taking care of the developmental issues of children aged 3–6 years in areas of cognitive, emotional, and social development	(CSP16) (PM12) (Aca37)
	Differences in executive structures at the provincial level	-Creating executive barriers due to lack of knowledge about ECD and executive structures of the State Welfare Organization and the Ministry of Education at the provincial level	(PM35)
	Weakness in referral and healthcare systems	-Problems in referring children with developmental disorders to second and third levels of healthcare due to lack of the implementation of a referral system -Healthcare system’s lack of capacity for screening children in all age ranges -Problems in the follow-up of children with developmental disorders -Failure to achieve the principles of community participation and intersectoral collaboration in PHC	(Aca24) (M17) (CSP33) (PM12)
4. **Factors affecting the evaluation of IECD policies**			
**Theme**	**Subtheme**	**Examples**	**Relation**
4.1- **Continuous and routine data registry system**	Lack of a comprehensive and integrated data registry system	-Lack of an integrated, accurate, and comprehensive database on different aspects of child development	(PM12)
	Superficiality of data collection mechanisms and insufficient research capacities	-Relying on available data and lack of surveys and case studies -Unreliability of available data	(PM32)
		-Impossibility of responding to children’s issues via single-field activities due to their complexity, variety, and comprehensiveness	(Aca 24)
		-Deficiencies in applied research in the field of children -Lack of interdisciplinary relationship in child-related research activities	(PM12) (PM35)
4.2- **Comprehensiveness of indexes**	Limiting IECD indexes to current indexes	-Lack of indexes in some areas and ignoring these indexes -Inadequate attention to distributive and justice-oriented indexes -Inadequate attention to qualitative indexes in different areas related to children.	(M 27) (PM13) (PM32)
	Lack of a reference body for formulating and designing comprehensive indexes	-Reporting a limited number of indexes by different organizations involved with children	(PM12)

1. **Agenda setting:** in this policy stage, three themes and seven sub-themes were identified.

### Problem stream

There are many problems in the field of children in developing countries, including our country, which inhibit the availability of equal opportunities for all children for optimal development. These problems exist in various areas such as early education and care, health and nutrition, and support. In addition to the existing problems in different areas, the lack of integration and coordination between policies and programs in different areas and the lack of focus on all aspects of children’s development as well as the lack of emphasis on eliminating existing inequalities have led to IECD policy.

*“Well, you can see there are various nutrition programs in the country but there are two points that have not been considered before the introduction of ECD. One is an integrated and comprehensive approach and the other is justice, both of which are very important in this policy. (PM13) “.*

### Politics stream

Numerous international political movements pursue the rights of children. Some of these international movements include the International Declaration on the Rights of the Child, the United Nations Convention on the Rights of the Child (CRC), Worst Forms of Child Labour Convention, the Universal Declaration of Human Rights, the United Nations International Covenant on Economic, Social and Cultural Rights (1966), the United Nations Millennium Development Goals (MDGs), United Nations Sustainable Development Goals (SDGs) adopted in 2015 for 2030 Agenda for Sustainable Development, Children’s Day, A World Fit For Children adopted by the UN General Assembly in 2002, emphasis on ECD in the WHO’s Commission on Social Determinants of Health (SDH), etc.

Following the formation of international political movements, national political movements emerged in this regard, including the Islamic Republic of Iran’s joining the Convention on the Rights of the Child, enacting laws related to the children’s rights including Law on Protection of Children and Adolescents adopted in 2003, establishing the Society for Protecting the Right of the Child in 1994, establishing the Committee on Social Determinants of Health and designating ECD as a priority, and referring to ECD in the Sixth Development Plan.

*“One of the most important subjects in social determinants of health is early childhood development. Why? Because interventions are far more effective if they start from early childhood. Second, there are recommendations for closing the gap in a generation that emphasize equity from the start. In prioritizing SDH domains, 14 priority subjects were determined, including ECD as a very important one. We started working on all 14 priorities, including ECD, resulting in the establishment of the corresponding secretariat in the Ministry of Health and designing a comprehensive document for it. I think it goes back to 2010-2011.” (PM8).*

### Policy stream

A key stage for formulating a national policy for IECD was to assemble committees in different fields to prepare reports on the children’s status and current policies in those fields. The corresponding committees were assembled in the fields of health, society, rights of the child, education, poverty and well-being, games, research, and special needs. Moreover, the IECD secretariat was established in the Ministry of Health to coordinate the activities between the main organizations involved in IECD. Moreover, sabbaticals were taken in other countries to use their experiences.

*“Well, in response to the concerns and interests of elites and key people in the field of children, presence of scientific and international evidence, and advances in reduced mortality and enhanced physical development of children, the policy stream has been created in Iran in the form of formulating the IECD document and establishing the corresponding secretariat. “(PM13).*

Our study showed that the three streams of problem, policy, and politics never came together and the window of policy opportunity has not been opend fully because the importance of the issues related to children is not understood sufficiently by policymakers at the highest levels of the country. Moreover, the attention paid to the problem stream at the middle levels in the three main organizations (the Ministry of Health and Medical Education, the Ministry of Education, and the State Welfare Organization) has resulted in the formation of policy stream only in the middle levels of these organisations.

2. **Policy formulation:** in this policy stage, three themes and six sub-themes were identified.

### Formulation and approval process

Another issue in this study was the long time it took to formulate the document and obtain its approval. In fact, its draft was prepared in 2012 and the primary approval was obtained from the Supreme Council of Health and Food Safety (SCHFS) in 2013. However, since a new government came to office that rescinded the approvals granted at the end of the previous government and changed the top managers of the three organizations involved in preparing the document, it took a long time to brief the new top managers and obtain the required approvals. For example, it took four years to brief the new managers on the subject.

*“The policy draft was finally prepared after a long time of about one year and a half. It was approved by the SCHFS on August 14, 2013 but in the next government, it became a matter of debate between the three involved organizations. Finally, it took 3-4 years until it was appended to the agenda of the committees of SCHFS and approved, and the executive mechanism of the document was approved in Moeen Council, which is presided by the Minister of Health and attended by deputy ministers,” (PM25).*

### Policy sustainability

Another point that was mentioned in the interviews as an important factor in decision making and policy formulation was unsustainability of policy as a result of changing the managers and its impact on the adopted approaches in relation to various issues.

*“The drawback of our structures is that contents are produced, but the next managers may not be committed to the contents and may have their own way around. These policies are not stable, nor are they sustainable and each manager designs and formulates policies from his or her own perspective,” (PM35).*

### Mechanisms of stakeholders’ participation in policymaking

The participation of stakeholders in policy design is one of the important issues which has a significant impact on the acceptance of formulated policies and leads to more appropriate policies. Two of the most important issues raised frequently in interviews with individuals from other organizations, including the Ministry of Education and the State Welfare Organization, who have worked with the Ministry of Health to develop the integrated early childhood development policy, were their dissatisfaction from the mechanism of participation and the inappropriate composition of the Supreme Council of Health Policy.

*“Intended participation in the form of a secretariat of the three main organizations involved in TECD policy is not a real participation, which does not seek help from our organizations in the production of content, and our participation is only for giving and receiving feedback.” (PM19).*

3. **Policy implementation:** in this policy stage, five themes and 12 sub-themes were identified. The results of this study showed that policy implementation was the most challenging step in the IECD policymaking process because this document has not yet been implemented at a national level although it was formulated several years ago. Its developers piloted the document in Takestan, Qazvin Province, and since they did not achieve their objectives, they formulated an executive protocol to implement it in Tehran, Kerman, and Kurdistan provinces as a pilot project. Moreover, public empowerment as part of the ECD project is being implemented in Chahar Dangeh, Eslamshahr, Tehran Province, by Vice Chancellery for Public Affairs of Tehran University of Medical Sciences through intersectoral collaboration.

### Conceptual ambiguity

The participants pointed to the problems resulting from the lack of consensus on the concept of ECD as one of the challenges and barriers in the implementation. Many individuals take a defensive stance towards it and consider it a concept contrary to the native culture of the country. To solve this problem, the name of the program has been changed to TAK, which is an abbreviation of it equivalent for early childhood development in Persian.

*“One of the main challenges is the term ECD itself because it is not a native and Persian term and some of the colleagues are sensitive to ECD without paying attention to its content. It has caused many problems in the implementation. It was supposed to be called TAK (Persian equivalent for ECD).” (PM19).*

### Intersectoral issues

In this category, the interviewees were faced with issues such as intersectoral competition, lack of teamwork and cross-sectoral collaboration culture, and non-binding cross-sectoral collaboration in the form of cross-sectoral agreements. Currently, each of the main organizations involved in IECD policy still pursue its own policies and programs separately and there is no integrity or coherence among these policies, which has resulted in wasting the resources, ignoring some domains, and even contrast between the current policies.

*“We only have a comprehensive and integrated view to the development of children in our policy design, and not in the implementation thereof. As for intersectoral collaboration, some actors in the organizations involved in this project do not feel so responsible and are more involved in organizational competitions.” (PM13).*

### Trans-sectoral issues

Lack of political commitment, lack of an institution or trans-sectoral structure to monitor and coordinate the child development program, and legal issues were the trans-sectoral issues mentioned in many interviews. For example, many interviewees stated that in the absence of a supreme institution or body for children, stakeholders would not be accountable for implementing ECD policies. However, some of the informants were against this idea and believed that this coordination could be done by one of the three main organizations involved in ECD.

*“Generally, in every country, an item receives attention if it is one of the priorities of the politicians and vice versa. Children and their related issues are not a priority for the country for the time being and there is no trace of ECD in public policymaking,” (CSP16).*

### Mobilization of resources

Problems in mobilizing resources, both human and financial resources, were repeatedly cited by interviewees as implementation challenges. With regard to human resources, the informants mentioned the shortage of skilled manpower in some related fields and the lack of empowerment of human resources due to the absence of a curriculum related to ECD in the education of child-related service providers. As to financial resources, they made mention of inadequate budget, improper budget allocation and lack of insurance coverage for some services like rehabilitation for developmental disorders, special diseases, counselling, etc. for children.

*“The role of pediatrician can be very important, but they only learn diagnosis and treatment of diseases while the promotion of development and even the treatment of developmental disorders are as non-pharmacological discussions.” (M14).*

*“One of the challenges is the lack of funding. In this regard, and in formulating the Sixth National Development Plan, we suggested the allocation of one percent of our GDP to ECD, but this was not approved. (PM39).”*

### Structural capacities

The structural issues mentioned in various interviews as one of the challenges in implementing the ECD program included the lack of necessary infrastructures for the development of children in health, education and other sectors, the differences in executive structures at provincial levels, the parallel structures, and the weakness in referral and healthcare systems.

*“The education sector is still unable to provide the safe and standard physical facilities in kindergartens and primary schools.” (PM12).*

*“We have a problem in referring children with developmental disorders to second and third levels of healthcare due to lack of an implemented referral system” (Aca24).*

4. **Policy evaluation:** in this policy stage, were identified two themes and four sub-themes were identified.

### Continuous and routine data registry system

According to the results of this study, there is no integrated mechanism for evaluating this program such that each sector is responsible for disseminating its indexes. There is no structure or mechanism for disseminating the data and indexes of the involved sectors in an integrated manner, for designing the required indexes. Nor is there such a mechanism for monitoring the data collection and dissemination in different organizations, and for applying the necessary modifications. According to the interviews, there is no data registry for children-related issues to disseminate the related indexes in different areas of education, health, laws, etc.

*“We should have a registry database. Every organization may have some data, for example, the Welfare Organization has the number of child laborers by nationality or age, but I have doubts about the validity of these data. I don’t know if these data are valid and reliable. How did they even find and count them? What I say is that we should have a complete data registry for different aspects of child development, similar to what we have for HIV/AIDS in the Ministry of Health. As for child abuse, dropout children, neglected children, and children’s educational status, I believe that the statistics, if there are any, are not valid and accurate.” (PM12).*

### Comprehensiveness of indexes

Another point that was referred to in various ways in the interviews was the lack of indexes in some areas and ignoring these indexes. Moreover, in the absence of national reference for designing comprehensive indexes, each organization has formulated a series of indexes in relation to its activity domain but these indexes lack comprehensiveness. For example, qualitative or distributive indexes are ignored in some areas.

*“In the field of education, the age group 4-6 years that is related to the policies made by Preschool Education Department, the coverage rate has increased by about 40%, i.e. it has increased from 32% to 70%; this growth has a large contribution to child development. However, special education indexes have not been evaluated. In other words, we have no indexes to determine where preschool children were and where they are now. We can only claim that we have increased the coverage rate, but it is not enough.” (PM32).*

## Discussion

Based on the results of our study, policymakers and actors related to IECD policy should consider a number of points to improve the policy process as follows:

### Developing strategic skills in policy community members to manage conflicts and choose effective participation mechanisms

Since policy formulation is the process of reaching agreements in several areas such as priority interventions, target groups, delivery mechanisms, institutional roles and responsibilities, leadership, and other important factors, this stage has a greater potential for conflicts compared to the agenda setting phase [21]. This conflict was also obvious in the present study since the results indicated that it has not been possible to achieve a holistic and integrated approach because of a delay in the formulation of policies due to lengthy administrative procedures and disputes among the main stakeholders. Also, there are problems and challenges among organizations involved in implementing ECD policies that hamper the implementation process. This can be resolved through creating strategic capacities in the policy community to manage conflicts and choose effective participation mechanisms. In this regard, Pelletier evaluated policy formulation for ECD in Bolivia, Peru, and Guatemala and reported that it is at the policy formulation stage when all the three countries faced contention over priority interventions, roles, responsibilities, and leadership and the weak interest and commitment of actors outside the health sector became evident. These conditions led to significant delays in the policy formulation process and incomplete implementation of the initially bold multi-sectoral vision, with the greatest progress seen in the health sector [21]. Also, Islam investigated the challenges of policy formulation and implementation for primary education in Bangladesh and found that the process of policy formulation was not performed properly due to the lack of democratic values and the influence of powerful elites in administration such that the interests of the civil society were ignored, making it difficult to achieve a common objective [22].

### Sustainability of designed policies and avoidance to change them after changing the managers

Instability of the policies and programs due to changes in governments and managers and their lack of commitment to previous policies and programs make it very difficult to implement long-term programs like ECD. In this regard, Islam found that implementing ECD policies has been unsuccessful in Bangladesh due to several factors including the political and cultural changes, the adoption of new decisions during the implementation process, and its removal from the political agenda by countries or supporting organizations [22].

### Necessity of a supreme institution or body for coordinating and supervising children’s affairs

In line with the results of this study, it is necessary to have an institute or ministry to coordinate the policies and activities related to ECD among various stakeholders and engaged organizations. Otherwise, consensus in policy design and intersectoral coordination in policy implementation will be difficult. For example, in a framework designed for ECD policymaking in Kenya, lack of a coordinating body or a leading ministry related to ECD was considered a challenge for implementing ECD policies [23].

### Political commitment and willingness in policymakers and high-level managers

Based on the results of the current study, the inclusion of ECD in the country’s macro agenda and its successful implementation requires that the policymakers be committed and have a desire to do so at the governmental level. To achieve this, we must rely on advocacy, negotiation and awareness-raising strategies. In this regard, Moyo reported that ignoring the importance of ECD on part of stakeholders and lack of a political will on the part of politicians were effective factors in the implementation of ECD programs [24]. Also, Bown studied factors affecting political decision-making processes and discussed the importance of public opinion, media pressure, and consultants and academics [25]. On the other hand, hindering a few factors such as the priorities of policy-makers and children’s voices have prevented the issue of macro policies from being adequately addressed in general and, as a result, ECD has remained a neglected subject in the country. In this line, Shawar et al. found that despite progress in generating priority for ECD, it is still a neglected issue, especially in low-income countries. Many reasons lead to insufficient priority for ECD in low-income countries, including scarce resources in these settings, poor understanding of its benefits, competing development priorities, and structures of inequality that hinder addressing the problems the poorest people face [26].

### Availability of information and indexes for various aspects of children’s development

According to the results of this study, it is not possible to have accurate, comprehensive, and valid statistics in the absence of an integrated and comprehensive data registry system about child abuse, child labor, dropout children, neglected children, educational status of children, etc. On the other hand, data collection problems result in contentment to the available routine data and lack of surveys and case studies, all raising the need for modification of the current data collection systems. In this regard, Islam found that the lack of institutionalization and the lack of coordination were the main problems in data collection [22]. Also, Pelletier discussed that the progress in protecting and improving ECD was constrained by multi-sectoral influences, weak or fragmented data, and monitoring systems as factors [21]. In this regard, the CDC states that while all evaluations face challenges, a large number of them can be overcome through designing proper indexes and methods [27]. Several studies have addressed the issues related to the evaluation of ECD policies, which are consistent with the results of the present study. For example, a report from Australia about Kids in Community Study (KiCS) discussed that due to challenges in relation to the complexity of different social contexts, it is difficult to develop strong social indexes for ECD (clear, measurable, and repeatable over time). To overcome this problem, mixed methods approaches (a combination of qualitative and quantitative data) should be used to provide a deep understanding of the society and the factors related to ECD outcomes [28].

### Promoting financial context and resources

Several studies have emphasized that the limitation of resources such as financial resources and skilled workforce and the unavailability of the required infrastructures are the challenges in implementing ECD policies. According to the results of a study by Boulle, the implementation of ECD policies is the least important element of the program mostly due to the nature of the segmented areas of the government, the issues related to organizational capacity, the lack of leadership, and the limitation of resources [29], which is consistent with the results of the current study. Based on our study, differences among different regions of the country should be eliminated in order to provide equal opportunities for all children to achieve their potential development.

## Conclusion

Early childhood development is a multi-sectoral and multidisciplinary subject with different aspects and layers, which is still neglected in Iran. Many efforts have been made to prioritize ECD in the SDH committee and to formulate an IECD policy as an integrated document which tries to engage the main organizations involved in ECD. It seems that ECD policymaking requires a deep belief in the importance of childhood in all levels of the society, especially in governmental levels. Despite the several challenges and barriers in each stage of the process of ECD policymaking, this process should be improved because better decisions and policies help to achieve better results in children’s welfare and health indexes, in reducing inequities, and in improving developmental outcomes. Otherwise, the conditions for equitable growth and development of children will not be provided and children will be deprived of their development potential, which will affect the sustainable development of the Iranian society considering the vital role of ECD in sustainable development.

### Strengths and limitations

This study was the first of its kind in conducting a deep and extensive analysis of ECD policies in Iran. The results of the current study can respond to many policy questions in this regard. However, our study has three main limitations; first, we have focused only on the policy process of ECD and have not explored the content and context of these policies. Second, we have not presented the developmental status of children in various areas of ECD in the form of figures due to the lack of statistics and information in this field in our country. Third, the data collection phase was delayed due to the busy schedule of the participants and their lack of time.

### Policy recommendations

The following items are the recommendations arising from our study that might contribute to improving the policy process of IECD:

- Mobilizing child-friendly media and groups, including child-friendly physicians, child-friendly lawyers, etc.
- Advocacy from high levels of government in connection with the importance of early childhood development policy.
- Strengthening inter-sectoral collaboration through incorporating it in the mission and evaluation of organizations, promoting commitment in the high levels of organizations, and developing an inter-ministerial policymaking framework that clearly specifies the roles and responsibilities of every sector and their interactions and collaborations.
- Promoting public awareness and support to maximize health and reduce potential remedial risk factors during pregnancy and after birth to 8 years of age.
- Developing applied and interdisciplinary research in the area of children to improve evidence-based policies.
- Designing appropriate intervention packages and programs to promote children’s development.
- Developing a universal service package for children and families.
- Explicit addressing of the issues and subjects related to ECD in the upstream documents of the country.
- Establishing parenting training and family empowerment programs.
- Prioritizing children in the welfare umbrella and paying attention to children in support of low-income families to reduce injustice.
- Promoting the quality of early childhood education services and emphasizing the training of life skills.
- Improving health programs in kindergartens and schools to meet the nutrition and physical activity needs of children in these environments.
- Empowering service providers in the field of children in connection with ECD.
- Improving screening and preventive interventions including screening for metabolic diseases and developmental disorders in children.
- Strengthening the existing executive infrastructures for optimal education of children.
- Designing a monitoring system in relation to various children’s affairs and creating a comprehensive data bank on various dimensions and areas of children’s development.
- Establishing an upstream institution or body such as the Children’s Commission in The Islamic Consultative Assembly or the Ministry of Children to coordinate, and conduct surveillance on, the affairs of children.
- Utilizing the capacity of NGOs and the private sector in policymaking and implementation.
- Reforming some laws and developing other laws with executive guarantees in order to protect children.

## Supplementary Information

**Additional file 1.**

## Data Availability

The data of this study are raw data which were accessible to the researchers in the interviews and are reported in the paper. The datasets used and/or analyzed during the current study are available from the corresponding author on reasonable request.
