# Supplementary material for: Integrated early childhood development policy in Iran: a qualitative policy process analysis
Source: BMC Public Health. 2021 Apr 2;21:649. doi: 10.1186/s12889-021-10646-7 (PMC8017628; doi:10.1186/s12889-021-10646-7)
Supplement: Supplementary file 1 — Additional file 1. [file 12889_2021_10646_MOESM1_ESM.pdf]

Integrated early childhood development policy in Iran: A Qualitative Policy Process Analysis

Omolbanin Atashbahar [1](PhD), Ali Akbari Sari (MD, PhD)[1,2], Amirhossein Takian [1,3,4] (MD, PhD), Alireza Olyaeemanesh [2,3](MD, PhD), Efat Mohamadi [3](PhD), Sayyed Hamed Barakati (MD)[5]

1. Department of Health Management and Economics, School of Public Health, Tehran University of Medical Sciences, Tehran, Iran
2. National Institute of Health Research, TehranUniversity of Medical Sciences, Tehran, Iran.
3. Health Equity ResearchCenter (HERC), TehranUniversity of Medical Sciences, Tehran, Iran.
4. Department of Global Health and Public Policy, School of Public Health, Tehran University of Medical Sciences, Tehran, Iran.
5. Population, Family and School Health Office, Ministry of Health and Medical Education, Tehran, Iran.

Omolbanin Atashbahar (Female): Email: o.atashbahar@gmail.com
Ali Akbari Sari(Male): Email: akbarisari@tums.ac.ir
Amirhossein Takian (Male): Email: takian@tums.ac.ir
Alireza Olyaeemanesh (Male): Email : arolyaee@gmail.com
Efat Mohamadi (Female): Email : efat.mohamadi@gmail.com
Sayyed Hamed Barakati(Male): Email : barekati h@health.gov.ir

Corresponding Author : Ali Akbari Sari, National Institute of Health Research, Tehran University of Medical Sciences, Tehran, Iran. Email: akbarisari@tums.ac.ir, Mobile: 09127251294, Tell: +982188992157. Address: National Institute of Health Research, No. 70, Bozorgmehr Ava., Vesal St., Keshavars Blvd., Tehran, Iran. Postal Code: 1416833481.

Appendix 1: Interview Guide

1- As an experienced person in this field, how do you assess the status of ECD in Iran? (physical, mental, cognitive, emotional, and social development)
2- What are the current ECD policies and programs in Iran? (any program or policy aiming at improving the children's capacity for development and learning in different levels like the child, family, and society or between different sectors like health, education, and social support)
3- Has there been a proper target setting in this regard, and does this target setting meet the current needs of the country?
4- To what extent have the above objectives been realized? What are your reasons?
5- When and why do you think ECD became one of the priorities of health policymaking?
6- Please explain the process and structure of formulating ECD policies and programs. How are they formulated and adopted?
7- Please explain the process and structure of implementing ECD policies and programs.
8- Please explain the process and structure of evaluating ECD policies and programs.
9- What macro and micro factors (including structural, situational, social, economic, political, and international factors) affect ECD policymaking in Iran?
10- What organizations, institutions, bodies, or persons are stakeholders in ECD? Please define their power, importance, role, effect, and interactions in this regard. Are they properly and adequately involved in the policymaking process?
11- If you had full authority in policymaking and making changes in this regard, what interventions did you give priority to in order to improve health, policymaking, and research?
12- What are your suggestions for improving inter-sectoral collaborations in this regard?
13- What are your suggestions for increasing the quality of these programs?
14- Are you aware of the IECD document that is now receiving the final approval in the High Council of Health?
15- What do you think about ICED document?
16- What changes or modifications (structural or other modifications) do you think is necessary for the ECD program to be successful?
17- Do you agree with the idea of the Ministry of Health acting as the coordinator and leader in this regard? Explain your reasons.

Appendix 2: Participants' characteristics

Interviewed Organization	Activity domains	Number of participants
Ministry of Health	Deputy of Social Affairs (SDH Administration, Social Harms Office,	3
	Population, Family, and School Health Office (Children's Health Office and Infants' Health Office)	3
	Ex manager of Nutrition Improvement Office	1
	Ex Minister	1
	Ex Health Deputy of Ministry of Health	1
State Welfare Organization of Iran	Deputy of Social Affairs	2
	Children and Adolescents' Affairs Office	1
	Ex Director of Children and Adolescents' Affairs Office	1
	Disability Prevention Office	1
Ministry of Education	Preschool Office	2
	Preschool Development and Planning Office	2
	Deputy of Physical Education and Health	1
Ministry of Cooperatives, Labour, and Social Welfare	Direct supports and compensatory activities, Collaboration in ECD project	1
Ministry of Interior	Deputy of Social Affairs, Collaboration in ECD project	1
University of Social Welfare and Rehabilitation Sciences	Member of Iranian Society of Pediatrics, Pediatrician, ex manager of Disability Prevention Office of State Welfare Organization of Iran	1
	Member of SDH Research Center	1
	Neonatologist, Member of Neonatal Development Committee of Ministry of Health	1
	Pediatrician, Member of Pediatric Neurorehabilitation Research Center, Collaboration in ECD project	1
Tehran University of Medical Sciences	Deputy of Social Affairs	1
	Pediatrician, Member of Iranian Society of Pediatrics	1
	Reproductive Health, Maternal and Child Health, Ex manager and executive officer in health centers of Ministry of Health	2
	Health policymaking and management	1
Judicial system of Iran	Judge	1
Institute for the Intellectual Development of Children and Young Adults	Cultural and Artistic Creations Center	1
	Research Deputy	1
DONYA Children's Research Institute	Member of Board of Directors	1
Society for Protecting the Rights of the Child (SPRC)	Member of Board of Directors	1

The Parliament of Iran	Support from Children and Young Adults Fraction	1
Ministry of Justice	National Body on the Convention on the Rights of the Child (CRC)	1
Tehran Municipality	Health Office	1
Children's Medical Center	Health service provider, Member of Growth and Development Research Center	1
High Council of Insurance	Secretariat of High Council of Health Insurance	1

Appendix 3: Information worksheet for document analysis

information worksheet		
1	Title	
2	Type	international law ⬭<br>Constitution ⬭<br>development plan ⬭<br>Other rules ⬭<br>Health policy and programs ⬭<br>Early care and education policy and program ⬭<br>Supportive policy and program ⬭
3	Date	
4	Source of design	
5	Materials	
6	Goals and values	
